# Single-session radiofrequency ablation versus microwave ablation of predominantly solid benign thyroid nodules—a comparison after propensity score matching for initial nodule volumes and diameters

**DOI:** 10.1007/s00330-025-11985-4

**Published:** 2025-09-12

**Authors:** Man Him Matrix Fung, Yan Luk, Brian Hung Hin Lang

**Affiliations:** Division of Endocrine Surgery, Department of Surgery, the University of Hong Kong, Queen Mary Hospital, Hong Kong, China

**Keywords:** Radiofrequency ablation, Microwave ablation, Thyroid nodule

## Abstract

**Objectives:**

The comparative efficacy of single-session radiofrequency ablation (RFA) versus microwave ablation (MWA) for benign solid thyroid nodules remains unclear because existing literature consists of heterogenous baseline and wide range of nodule volumes. This study identified the predictors of volume reduction rate (VRR) of RFA versus MWA, and compared the efficacy between the two treatments.

**Materials and methods:**

Consecutive benign nodules ≥ 80% solid, treated with a single session of RFA or MWA in a tertiary endocrine surgery center, with at least 12-month follow-up, were included. Propensity score matching (PSM) for baseline characteristics was performed to compare RFA vs MWA. The primary outcome was 12-month VRR.

**Results:**

From 2021 to 2023, 208 nodules were analyzed (RFA: 142, MWA: 66). Maximum nodule diameter and initial nodule volume correlated with 12-month VRR (*p* < 0.001, correlation coefficient −0.360 and −0.322, respectively) of RFA but not MWA. PSM analysis for age, sex and maximum nodule diameter; or age, sex and initial nodule volume showed comparable overall 12-month VRR between RFA and MWA. In subgroup analysis, MWA achieved greater 3-month and 12-month (78.0% ± 15.5 vs 67.3% ± 17.6, *p* = 0.028) VRR for nodules with a maximum diameter ≥ 3.5 cm; and greater 6-month VRR for nodules with volume ≥ 20 mL (71.4% ± 16.7 vs 57.2% ± 16.1, *p* = 0.030). No significant complications occurred in either ablation modality.

**Conclusion:**

Larger nodule diameter and volume negatively correlated with the treatment efficacy of single-session RFA, but not MWA. Single-session MWA achieved greater VRR for nodules with maximum diameter ≥ 3.5 cm, or volume ≥ 20 mL. The results of this study prompt confirmation by future randomized controlled trials with stratification by nodule volume.

**Key Points:**

***Question*** *The comparative efficacy of single-session radiofrequency ablation (RFA) versus microwave ablation (MWA) for benign solid thyroid nodules remains unclear and so are factors affecting treatment efficacy*.

***Findings*** *Larger nodule diameter and volume negatively correlated with efficacy of RFA. For smaller nodules, RFA and MWA were comparable. MWA may have better efficacy for larger nodules*.

***Clinical relevance*** *For smaller nodules (max diameter < 3.5* *cm or volume < 20* *mL), RFA and MWA were equally effective and safe. For larger nodules (max diameter ≥ 3.5* *cm or volume ≥ 20* *mL), MWA may be more efficacious. Verification with prospective trials that account for baseline nodule volumes is needed*.

**Graphical Abstract:**

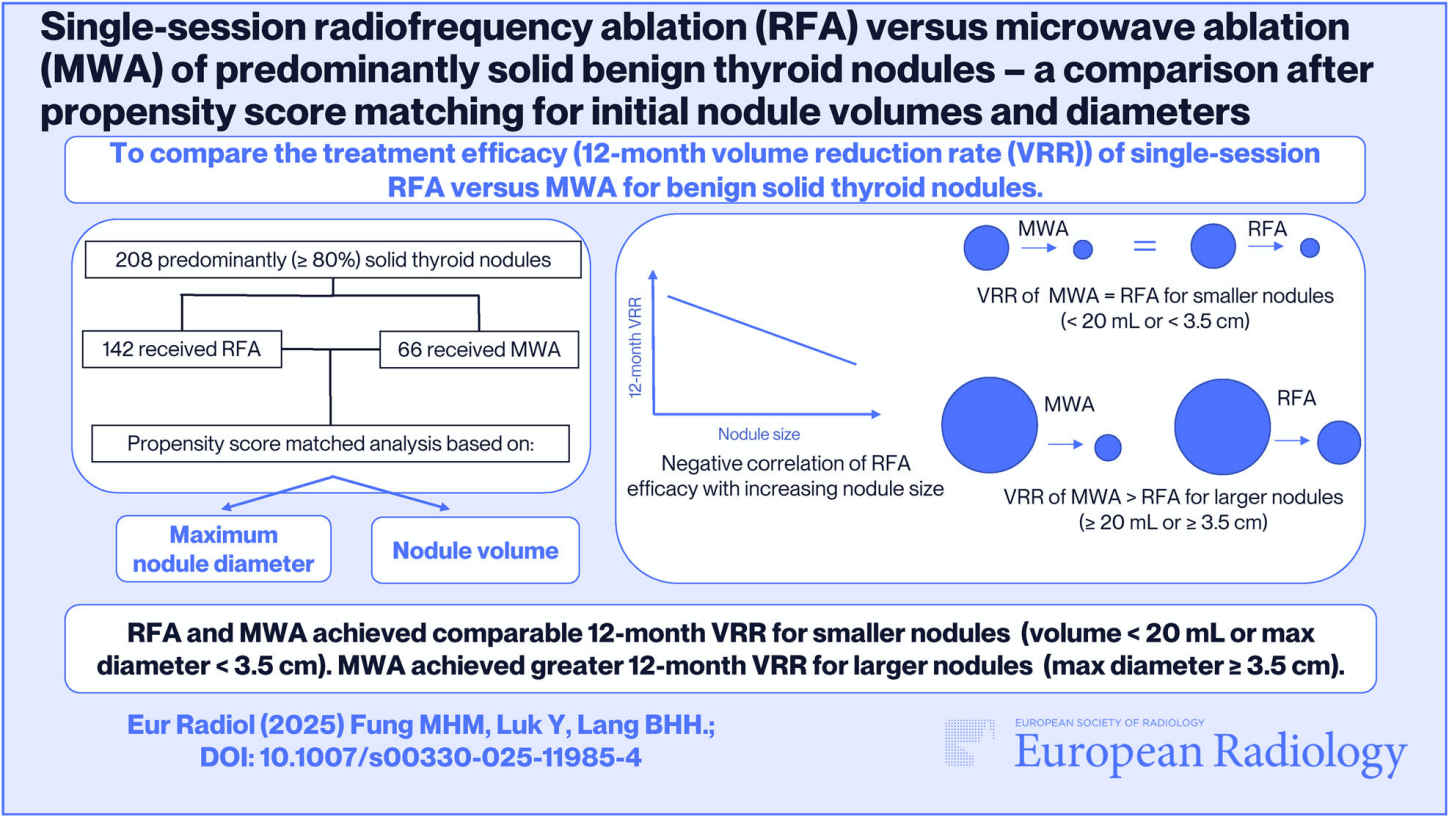

## Introduction

Thyroid nodules affect up to 60% of the population [1]. Up to 10–15% benign thyroid nodules can cause compressive symptoms, including neck pressure, difficulty swallowing or breathing, or cosmetic concerns. Ultrasound-guided percutaneous thermal ablation is an effective, minimally invasive treatment for symptomatic benign thyroid nodules [2–6]. Among various thermal ablation modalities, radiofrequency ablation (RFA) and microwave ablation (MWA) were the two commonly used to treat benign thyroid nodules. RFA uses radiofrequency waves to agitate ions around the electrode, creating frictional heat to achieve ablation [4, 5]. The efficacy of RFA can be limited by the heat sink effect, changes in tissue impedance during ablation, and long procedure times. On the other hand, MWA uses electromagnetic energy from microwaves to induce thermal ablation around the antenna [7–10]. It is less affected by the heat sink effect and impedance changes during ablation, and may lead to shorter treatment duration.

Given the fundamental differences in the mechanism of heat generation between RFA and MWA, and different susceptibility to the heat sink effect, there may be potential differences in the treatment efficacy between the two. However, the current evidence comparing RFA versus MWA in thyroid ablation was limited and conflicting [11–16]. Fair comparison was often limited by unmatched and heterogeneous baseline nodule volumes and characteristics [11–13]. Most importantly, from the clinical perspective, the most influential clinical factors on treatment efficacy for each modality, which would guide the choice of ablation modality, remain unknown. In light of such, the current study aims to first identify the factors that affect the treatment efficacy of single-session RFA and single-session MWA individually for predominantly solid benign thyroid nodules; and second, to compare the treatment efficacy, i.e., 12-month volume reduction rate (VRR) between single-session RFA and MWA, after propensity score matching of the factors identified in the first step.

## Materials and methods

This study followed the principles in the Declaration of Helsinki and was approved by the Institutional Review Board of the University of Hong Kong/Hospital Authority Hong Kong West Cluster (UW 24-566). Since this was a retrospective study, patients’ consents were waived.

Consecutive patients undergoing single-session thermal ablation from a tertiary endocrine surgery unit were analyzed. To be eligible, patients have to be (1) adults aged ≥ 18 years, (2) have symptomatic and predominantly solid (≥ 80% solid) thyroid nodule(s) that were confirmed benign (Bethesda class II) by fine needle aspiration cytology, (3) underwent a single session of RFA or single-session MWA within a 12-month period, (4) completed follow-up of 12 months after RFA or MWA. Nodules were excluded if they (1) received more than one session of ablation within 12 months, (2) received previous thermal ablation treatment (e.g., RFA, MWA or high-intensity focused ultrasound), (3) contained > 10% coarse calcification as shown on ultrasound, (4) toxic thyroid nodule as evidenced by thyroid scintigraphy.

### Procedure of thermal ablation

The tertiary endocrine surgery unit has been performing thyroid RFA for 5 years and MWA for 2 years. All ablations were performed by two endocrine surgeons, each with the experience of more than 100 cases of thyroid RFA procedures and 50 cases of thyroid MWA procedures, respectively. Each of the two surgeons had a comparable number of ablations performed for the current cohort (*p* = 0.808) (Supplementary Table 1). Until late 2022, microwave ablation was not available and therefore only RFA was performed before this period. After late 2022, nodules received either RFA or MWA based on the availability of the machine.

For RFA, the same radiofrequency generator (STARmed VIVA RF generator) and electrode (star RF Electrode—Fixed, 18 G 70 mm (electrode length), 10 mm (active tip length)) were used. For MWA, the same MWA generator (ECO, Nanjing) and electrode (ECO1007, 17 G 3 mm) were used. Ablation power was set to the level recommended by the manufacturer (50–60 W for RFA; 30 W for MWA).

All ablations were done under intravenous sedation and peri-thyroidal local anesthesia [17]. The trans-isthmic approach and moving shot technique were applied [4]. In short, the target nodule was divided into imaginary transverse planes, each constituting imaginary subunits. The electrode was typically placed from deep to superficial, and from lateral to medial at the start of ablation. Each subunit will be considered successfully ablated when microbubbles are observed at the site of ablation; afterward, the electrode will be moved to ablate the next subunit. The entire transverse plane was considered ablated completely when it was entirely filled with microbubbles. The ultrasound transducer and ablation needle were then advanced to the next imaginary transverse plane. The danger triangle was avoided. Intra-operative laryngeal ultrasonography of the vocal cords was used to monitor vocal cord function throughout the procedure [18]. Also, the patient’s heart rate, blood pressure, respiration rate, and peripheral oxygenation were monitored. Peri-operative adverse events were recorded. The patients were observed for 4–6 h after the procedure and discharged when they had recovered from the effects of sedation.

### Correlation analysis and propensity score matching

Electronic health records were reviewed, and factors that correlate with 12-month VRR for each of the ablation methods, i.e., RFA and MWA, were evaluated. Tables 1 and 2 showed the factors under consideration, which included patient baseline characteristics (age, sex, body mass index, serum TSH, FT4, thyroglobulin, anti-thyroglobulin, anti-thyroid peroxidase), nodule baseline characteristics (volume, percentage solid component), and ablation parameters (energy applied per unit volume, ablation time per unit volume). After correlation analysis, maximum nodule diameter and initial nodule volume were noted to have significant correlation with 12-month VRR  (Table 2). Therefore, analysis was performed after propensity score matching for (1) age, sex and maximum nodule diameter; and (2) age, sex and initial nodule volume. Propensity scores were estimated by the logistic regression model, followed by nearest-neighbor matching. Ablation energy parameters were not used for propensity score matching, because (1) RFA and MWA were considered distinct modalities with different mechanisms of heat generation, (2) Ablation parameters did not correlate with 12-month VRR (Table 2) and (3) The endpoint of ablation for both techniques is primarily determined by intra-operative ultrasound findings when the entire nodule appears filled with microbubbles, rather than a fixed energy or ablation time goal.

**Table 1:** Baseline characteristics of thyroid nodules that were included for analysis (*n* = 208)

	Overall *n* = 208
Age	52 (45–60)
Sex (M:F)	11:151
RFA:MWA	142:66
Number of nodules ablated at the same session, *n* (%)	
1	115 (55)
2	72 (35)
3	21 (10)
Pre-ablation width (cm)	2.74 ± 1.05
Pre-ablation depth (cm)	2.25 ± 0.88
Pre-ablation height (cm)	3.11 ± 1.22
Pre-ablation maximum diameter (cm)	3.34 ± 1.19
Pre-ablation volume (mL)	12.1 ± 12.2
Solid component (%)	100 (100–100)
TSH (mIU/L)	0.97 (0.50–1.42)
FT4 (pmol/L)	17 (15–18)
Thyroglobulin (U/L)	77 (26–219)
Anti-thyroglobulin raised^	33
Anti-thyroid peroxidase antibody raised^	32
Total energy delivery (J)	17,405 (8954–38,660)
Ablation time (s)	578 (261–1041)
Energy per unit volume (J/mL)	2281 (1500–3188)
Ablation time per unit volume (s/mL)	61 (41–85)
3-month VRR (%)	58.5 ± 20.3
6-month VRR (%)	67.2 ± 18.9
12-month VRR (%)	74.4 ± 17.9
Same-day discharge (%)	100
Hematoma requiring re-admission	0
Vocal cord palsy	0

^ Value > 100 U/L

**Table 2:** (A) Correlation analysis with 12-month volume reduction rate after single-session radiofrequency ablation (*n* = 142); (B) Correlation analysis with 12-month volume reduction rate after single-session microwave ablation (*n* = 66)

Factor	Correlation coefficient (95% CI)	*p*-value
**A**		
Age	0.097 (−0.074 to 0.262)	0.251
Body mass index	−0.014 (−0.236 to 0.210)	0.904
Pre-ablation		
TSH	0.004 (−0.183 to 0.192)	0.192
FT4	−0.032 (−0.221 to 0.159)	0.733
Thyroglobulin	0.024 (−0.176 to 0.222)	0.807
Anti-thyroglobulin antibody	−0.070 (−0.250 to 0.116)	0.462
Anti-thyroid peroxidase antibody	−0.114 (−0.294 to 0.074)	0.235
Maximum nodule diameter	**−0.360 (−0.495 to −0.208)**	**< 0.001**
Initial nodule volume	**−0.317 (−0.457 to −0.160)**	**< 0.001**
Nodule solid component	0.046 (−0.119 to 0.210)	0.583
Energy per unit volume	−0.072 (−0.235 to 0.095)	0.400
Time per unit volume	0.111 (−0.064 to 0.279)	0.213
**B**		
Age	−0.218 (−0.443 to 0.032)	0.078
Body mass index	−0.189 (−0.464 to 0.120)	0.214
Pre-ablation		
TSH	0.157 (−0.124 to 0.414)	0.258
FT4	0.022 (−0.252 to 0.293)	0.872
Thyroglobulin	0.025 (−0.272 to 0.318)	0.867
Anti-thyroglobulin	0.077 (−0.203 to 0.345)	0.581
Anti-thyroid peroxidase antibody	0.191 (−0.089 to 0.443)	0.167
Maximum nodule diameter	−0.130 (−0.361 to 0.115)	0.297
Initial nodule volume	−0.123 (−0.361 to 0.130)	0.326
Nodule solid component	0.126 (−0.127 to 0.363)	0.315
Energy per unit volume	0.283 (−0.122 to 0.379)	0.283
Time per unit volume	0.130 (−0.123 to 0.367)	0.297

Bold = statistically significant (*p* < 0.05)

### Outcomes

The primary outcome was the volume reduction rate (VRR) at 12 months after ablation. The same ultrasound machine (Samsung HS30, Korea) and transducer (Samsung LN5-12, Korea) were used for nodule volume measurements. Nodule volume was calculated with the formula of width × depth × length (in cm) × 0.523 [19]. Volume reduction rate (VRR) was calculated by the formula: ([Vol_basal_ − Vol_final_] · 100)/Vol_basal_ [4, 20]. Nodule measurements were performed by the same endocrine surgeon at all time points. Intra-observer variability was assessed by repeating measurements of nodule dimensions before ablation (baseline) in 1 week for 40 nodules. The intra-class correlation coefficient (ICC) for both nodule diameters and volume measurements was excellent (0.996–0.999). Detailed reliability results were presented in Supplementary Table 2.

The percentage of the solid component of nodules was determined with ultrasound, with anechoic areas considered as non-solid, and hypo-, iso- and hyperechoic areas considered as solid areas. The secondary outcomes included 3-month VRR, 6-month VRR, early and late complications. Flexible laryngoscopy was used to evaluate vocal cord mobility on day 7 after ablation. Cosmetic symptoms score was recorded with a 1–4 numerical scale (1 being no palpable goiter, 4 being easily visible goiter), and compressive symptoms score was recorded with a 0–100 numerical scale (0 being no compressive symptoms and 100 being the most severe compressive symptoms) [20].

### Statistical analysis

Spearman correlation analysis was used to identify factors associated with 12-month VRR separately for RFA and MWA. Propensity score-matched analysis was performed as mentioned above for the comparison of 12-month VRR. For baseline variable comparison, Student’s two-tailed *t*-test was used for variables with normal distribution and Mann–Whitney U test was used for non-parametric data. Comparison of categorical variables was performed by means of the χ^2^ test. Statistical analysis was performed with IBM SPSS version 28. A *p*-value of < 0.05 will be considered statistically significant.

## Results

**Fig. 1:**
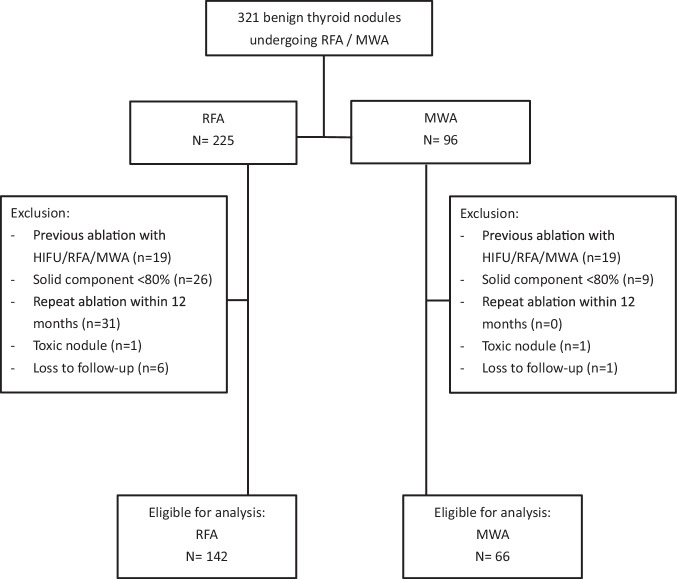
Study flow chart. From 2021 to 2023, 321 benign thyroid nodules underwent radiofrequency ablation and microwave ablation. After exclusion, 208 predominantly solid nodules were included for analysis

From 2021 to 2023, 321 benign thyroid nodules received RFA or MWA (Fig. 1). After excluding patients with previous ablation (*n* = 38), solid component < 80% (*n* = 35), having repeat ablation within 12 months (*n* = 31), toxic nodule (*n* = 2), and loss to follow-up (*n* = 7), 208 benign nodules from 160 patients were included for the study (RFA = 142, MWA = 66). Baseline characteristics and ablation parameters are shown in Table 1. The median age was 52 (45–60), and 151 (93%) were female patients. The mean nodule volume was 12.2 ± 12.2 mL. The median percentage solid component of nodules was 100% (90–100%). None of the patients received previous thermal ablation. All patients had normal pre-operative TSH and FT4. The mean energy delivered per unit volume was 2281 J/mL (1500–3188), and the median ablation time per unit volume was 61 s/mL (41–85). Overall, the 3-month VRR was 58.5 ± 20.3%, the 6-month VRR was 67.2 ± 18.9%, and the 12-month VRR was 74.4 ± 17.9%. All patients were discharged on the same day after ablation, and none suffered from hematoma requiring re-admission or vocal cord palsy.

**Fig. 2:**
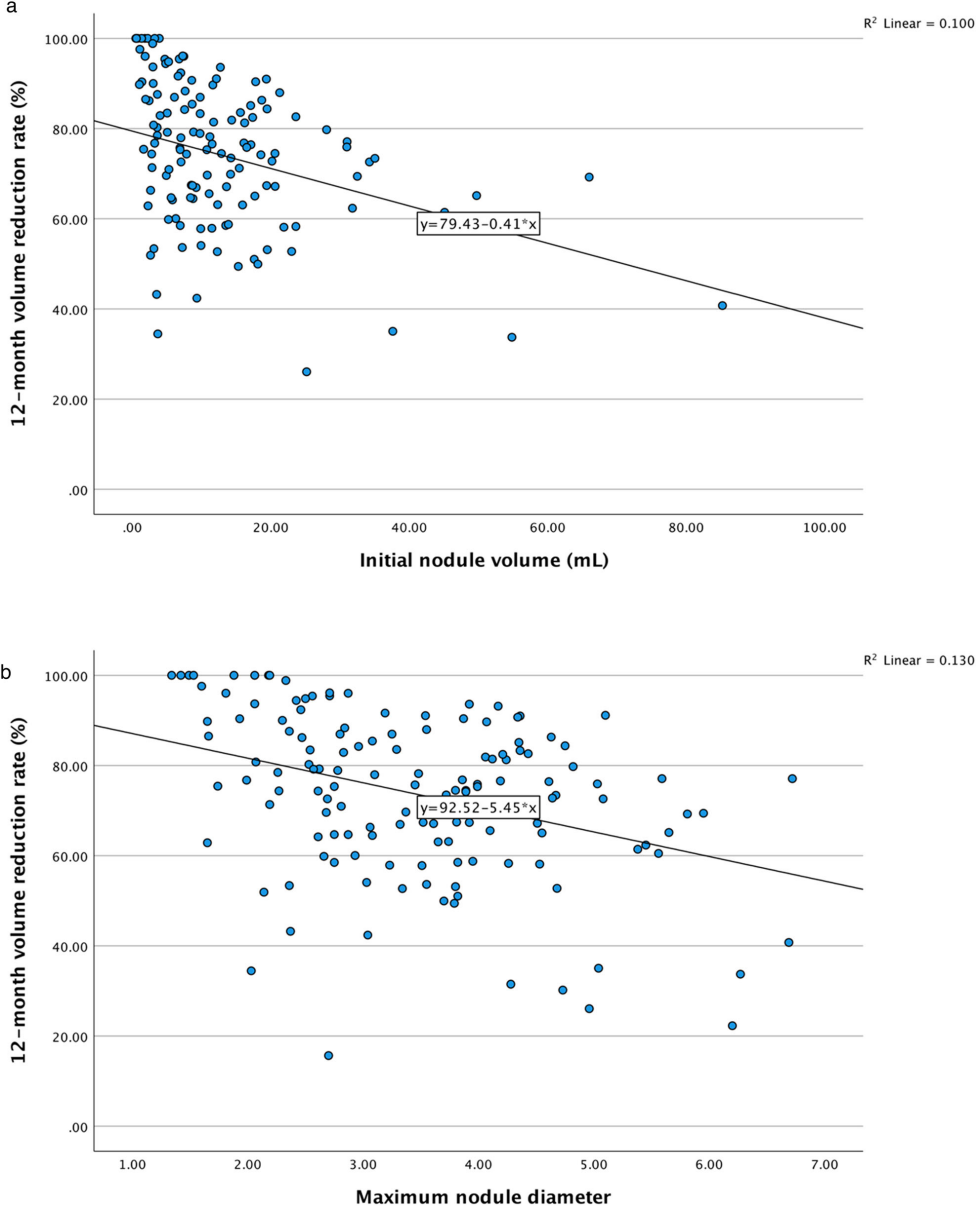
**a** Scattered plot of 12-month volume reduction rate against initial nodule volume after single-session radiofrequency ablation of benign, predominantly solid thyroid nodules. A negative correlation was observed (correlation coefficient −0.317 (−0.457 to −0.160), *p* < 0.001). **b** Scattered plot of 12-month volume reduction rate against maximum nodule diameter after single-session radiofrequency ablation of benign, predominantly solid thyroid nodules. A negative correlation was observed (correlation coefficient −0.360 (−0.495 to −0.208), *p* < 0.001)

The factors that correlated with 12-month VRR of RFA and MWA were shown in Table 2A, B. For RFA (Table 2A and Fig. 2a, b), maximum nodule diameter and initial nodule volume negatively correlated with 12-month VRR significantly (correlation coefficient −0.360 (−0.495 to −0.208), *p* < 0.001 and −0.322 (−0.476 to −0.150), *p* < 0.001, respectively). For MWA (Table 2B), maximum nodule diameter and initial nodule volume did not affect 12-month VRR, nor did the other factors under consideration. For nodules receiving RFA, those with maximum diameter ≥ 3.5 cm (75th percentile of our cohort’s maximum diameter) had lower 12-month VRR than those < 3.5 cm (68.8% ± 17.3 vs 78.2% ± 17.7, *p* = 0.002) (Supplementary Table 3a). Similarly, nodules with initial volume ≥ 20 mL (75th percentile of our cohort’s nodule volume) had lower 12-month VRR than those < 20 mL after RFA (64.4% ± 20.4 vs 75.9% ± 16.7, *p* = 0.002). Whereas for nodules receiving MWA, no significant differences in 3-month, 6-month or 12-month VRR were observed between larger and smaller nodules using the same cut-offs for maximum diameter or volume (Supplementary Table 3b).

Based on the above findings that maximal nodule diameter and initial nodule volume were the only two factors that significantly influenced RFA outcomes, PSM analysis was after matching of propensity scores of (1) age, sex and maximum nodule diameter, and (2) age, sex and initial nodule volume (Table 3A, B). The baseline characteristics of RFA and MWA groups are shown in Table 3A. Before matching, significant differences in initial nodule volume and maximum nodule diameter were noted between the RFA and MWA groups. After 1:1 propensity score matching for each respective analysis, the baseline maximum nodule diameter and initial nodule volume were balanced (*p* > 0.05). No significant differences were found between groups regarding demographics, body mass index, solid component of nodules, additional nodule ablation in the same session, baseline thyroid function, thyroglobulin and thyroid antibodies (*p* > 0.05). Table 3B compared the ablation results. Comparable 12-month VRR were noted between RFA and MWA by 1:1 PSM either for age, sex and maximum nodule diameter; or age, sex and initial nodule volume (74.4% ± 19.5 vs 77.3% ± 17.4, *p* = 0.372 and 76.9% ± 15.9 vs 77.3% ± 17.4, *p* = 0.883, respectively). MWA required shorter total ablation time, shorter ablation time per unit volume, less total energy delivery, and less energy per unit volume delivery than RFA (*p* < 0.05). All patients had improvement in compressive and cosmetic symptoms, with no differences in the degree of improvement between the two groups.

**Table 3:** (A) Propensity score-matched (PSM) analysis comparing radiofrequency ablation versus microwave ablation—comparison of baseline characteristics before and after PSM for age, sex and maximum nodule diameter and PSM for age, sex and initial nodule volume; (B) Propensity score-matched (PSM) analysis comparing radiofrequency ablation versus microwave ablation—comparison of ablation outcomes before and after PSM for age, sex and maximum nodule diameter and PSM for age, sex and initial nodule volume

	Before propensity score matching			After 1:1 propensity score matching for age, sex and maximum nodule diameter			After 1:1 propensity score matching for age, sex and initial nodule volume		
	RFA (*n* = 142)	MWA (*n* = 66)	*p*-value	RFA (*n* = 66)	MWA (*n* = 66)	*p*-value	RFA (*n* = 66)	MWA (*n* = 66)	*p*-value
**A**									
Age	54 (46–62)	51 (44–57)	0.103	50 (45–58)	51 (44–57)	0.902	53 (42–60)	51 (44–57)	0.738
Sex (M:F)	10:132	5:61	> 0.999	3:63	5:61	0.718	5:61	5:61	> 0.999
Body mass index	23.2 ± 3.6	23.7 ± 4.7	0.466	22.5 ± 3.3	23.7 ± 4.7	0.150	23.7 ± 3.9	23.7 ± 4.7	0.978
Initial nodule volume (mL)	14.1 ± 13.8	10.3 ± 11.5	**0.049**	11.1 ± 14.2	10.3 ± 11.5	0.720	10.4 ± 10.4	10.3 ± 11.5	0.924
Pre-ablation maximum diameter (cm)	3.48 ± 1.19	3.05 ± 1.14	**0.015**	3.06 ± 1.21	3.05 ± 1.14	0.933	3.1 ± 1.1	3.0 ± 1.1	0.755
Solid component (%)	100 (100–100)	100 (90–100)	0.830	100 (100–100)	100 (90–100)	0.621	100 (100–100)	100 (90–100)	0.621
> 1 nodule ablated	63	30	0.636	30	30	> 0.999	30	30	> 0.999
Baseline									
TSH	0.87 (0.44–1.42)	1.07 (0.70–1.44)	0.090	1.00 (0.49–1.63)	1.07 (0.70–1.44)	0.558	0.86 (0.45–1.51)	1.07 (0.70–1.44)	0.277 0.051
FT4	17 (15–19)	17 (16–18)	0.099	17 (15–19)	17 (16–18)	0.164	17 (15–19)	17 (16–18)	0.905
Thyroglobulin	67 (23–240)	95 (27–219)	0.584	53 (26–240)	95 (27–219)	0.522	90 (40–221)	95 (27–219)	0.648
Anti-TPO positive*	28	5	0.180	12	10	0.812	12	10	0.812
Anti-Tg positive*	27	5	0.177	12	7	0.318	12	7	0.318
**B**									
3-month VRR (%)	58.5 ± 20.3	57.0 ± 20.5	0.164	56.8 ± 21.2	62.2 ± 19.5	0.213	60.7 ± 20.7	62.2 ± 19.5	0.723
6-month VRR (%)	65.3 ± 19.3	70.8 ± 17.9	0.075	66.0 ± 18.9	70.8 ± 17.9	0.186	66.8 ± 19.9	70.8 ± 17.9	0.286
12-month VRR (%)	73.6 ± 18.1	77.3 ± 17.4	0.164	74.4 ± 19.5	77.3% ± 17.4	0.372	76.9 ± 15.9	77.3 ± 17.4	0.883
Ablation time (s)	775 (345–1213)	345 (186–577)	**< 0.001**	644 (244–1084)	345 (186–577)	**0.002**	600 (220–1166)	345 (186–577)	**0.009**
Total energy delivery (J)	28,242 (11,213–50,867)	10,290 (5640–17,280)	**< 0.001**	16,778 (8887–37,990)	10,290 (5640–17,280)	**0.002**	17,321 (7448–41,788)	10,290 (5640–17,280)	**0.002**
Energy per unit volume (J/mL)	2539 (1957–3528)	1568 (987–2265)	**< 0.001**	2672 (2056–4061)	1568 (987–2265)	**< 0.001**	2586 (2128–3502)	1568 (987–2265)	**< 0.001**
Ablation time per unit volume (s/mL)	63 (48–91)	53 (33–74)	**0.015**	65 (50–112)	53 (33–74)	**0.005**	69 (49–125)	53 (33–74)	**0.007**
Compressive symptoms score									
Baseline	35 (10–50)	40 (10–50)	0.658	40 (10–52.5)	40 (10–50)	0.553	30 (12.5–52.5)	40 (10–50)	0.895
12-month	5 (0–20)	7.5 (0–20)	0.838	5 (0–20)	7.5 (0–20)	0.725	0 (0–20)	7.5 (0–20)	0.387
Cosmetic symptoms score									
Baseline	4 (2.75–4)	4 (3–4)	0.497	4 (2–4)	4 (3–4)	0.971	4 (2–4)	4 (3–4)	0.635
12-month	2 (1–4)	1 (1–3)	< 0.001	2 (1–4)	1 (1–3)	0.004	2 (1–3)	1 (1–3)	0.118

Bold = statistically significant (*p* < 0.05)

* > 100 U/L

Subgroup analyses were conducted to evaluate how maximum nodule diameter and initial nodule volume affected the comparative efficacy of single-session RFA versus MWA. For nodules with maximum diameter ≥ 3.5 cm (≥ 75th percentile), after 1:1 PSM, MWA led to greater 3-month (65.0% ± 19.3 vs 50.0% ± 17.0, *p* = 0.030) and 12-month VRR (78.0% ± 15.5 vs 67.3% ± 17.6, *p* = 0.028) than RFA (Table 4A, B). For nodules with maximum diameter < 3.5 cm, no differences in 12-month VRR were observed between MWA and RFA (77.0% ± 18.2 vs 77.5% ± 19.7, *p* = 0.907) (Supplementary Table 4a, b).

**Table 4:** (A) Subgroup analysis of nodules with maximum diameter ≥ 3.5 cm, comparison of baseline characteristics before and after propensity score matching for age, sex and maximum nodule diameter; (B) Subgroup analysis of nodules of nodules with maximum diameter ≥ 3.5 cm, comparison of ablation outcomes before and after propensity score matching for age, sex and maximum nodule diameter

	Before propensity score matching			After 1:1 propensity score matching		
	RFA (*n* = 70)	MWA (*n* = 18)	*p*-value	RFA (*n* = 18)	MWA (*n* = 18)	*p*-value
**A**						
Age	53 (45–60)	52 (46–57)	0.422	50 (44–58)	52 (46–57)	0.702
Sex (M:F)	3:67	2:16	0.270	0:18	2:16	> 0.999
BMI	23.1 ± 3.8	24.1 ± 3.7	0.439	20.7 ± 2.0	24.1 ± 3.7	**0.006**
Nodule volume (mL)	23.2 ± 15	24.8 ± 13.3	0.680	25.0 ± 19.4	24.8 ± 13.3	0.965
Maximum diameter	4.46 ± 0.81	4.52 ± 0.83	0.780	4.52 ± 0.99	4.52 ± 0.83	0.999
> 1 nodule ablated (%)	28	5	0.392	5	5	0.215
Baseline:						
TSH	0.82 (0.46–1.30)	1.01 (0.50–1.67)	0.346	0.82 (0.32–1.23)	1.01 (0.50–1.67)	0.361
FT4	17 (15–18)	17 (16–18)	0.690	18 (15–19)	17 (16–18)	0.545
Thyroglobulin	57 (21–214)	172 (51–248)	0.195	52 (18–224)	172 (51–248)	0.239
Anti-TPO positive*	16	5	0.151	4	5	> 0.999
Anti-Tg positive*	12	2	0.431	2	2	> 0.999
Solid component (%)	100 (90–100)	97 ± 7	0.243	100 (90–100)	100 (100–100)	0.272
**B**						
3-month VRR (%)	56.4 ± 16.2	65.0 ± 19.3	**0.049**	50.0 ± 17.0	65.0 ± 19.3	**0.030**
6-month VRR (%)	64.9 ± 16.1	71.4 ± 15.2	0.078	62.9 ± 18.3	71.4 ± 15.2	*0.083*
12-month VRR (%)	68.8 ± 17.3	78.0 ± 15.5	**0.022**	67.3 ± 17.6	78.0 ± 15.5	**0.028**
Procedural time (s)	1124 (771–1611)	761 (599–1197)	**0.020**	1019 (762–1910)	761 (599–1197)	0.064
Total energy delivery (J)	49,329 (32,112–75,584)	22,020 (17,940–35,910)	**< 0.001**	48,680 (30,324–96,316)	22,020 (17,940–35,910)	**< 0.001**
Energy per unit volume (J/mL)	2463 (1808–3186)	987 (830–1127)	**< 0.001**	2534 (1757–3134)	987 (830–1127)	***< 0.*0*01***
Ablation time per unit volume (s/mL)	57 (43–71)	33 (26–39)	**< 0.001**	55 (45–67)	33 (26–39)	**< 0.001**
Compressive symptoms score						
Baseline	30 (10–50)	10 (0–50)	0.179	30 (5–48)	10 (0–50)	0.492
12-month	7.5 (0–30)	10 (0–22.5)	0.560	8 (0–30)	10 (0–22.5)	0.616
Cosmetic symptoms score						
Pre-ablation	4 (4–4)	4 (2.3–4)	0.373	4 (4–4)	4 (3–4)	0.351
Post-ablation	3 (2–4)	3 (2–3.5)	0.237	3 (2–4)	3 (1–3)	0.128

*VRR* volume reduction rates

Bold = statistically significant (*p* < 0.05)

* 100 U/L

Similarly, for nodules with initial volume ≥ 20 mL (≥ 75th percentile), after 2:1 PSM, MWA led to significantly greater 6-month VRR than RFA (71.4% ± 16.7 vs 57.2% ± 16.1, *p* = 0.030) (Table 5A, B). Numerically greater 12-month VRR was also observed for the MWA group, albeit being statistically insignificant. For nodules with initial volume < 20 mL, after 1:1 PSM, no significant differences were observed in 12-month VRR between MWA and RFA (78.3% ± 17.1 vs 75.8% ± 18.5, *p* = 0.470) (Supplementary Table 5a, b)

**Table 5:** (A) Subgroup analysis of nodules with initial volume ≥ 20 mL, comparison of baseline characteristics before and after propensity score matching for age, sex and initial nodule volume; (B) Subgroup analysis of nodules with initial volume ≥ 20 mL, comparison of ablation outcomes before and after propensity score matching for age, sex and initial nodule volume

	Before propensity score matching			After 2:1 propensity score matching		
	RFA (*n* = 29)	MWA (*n* = 9)	*p*-value	RFA (*n* = 18)	MWA (*n* = 9)	*p*-value
**A**						
Age	51 (42–59)	53 (48–57)	0.853	53 (41–63)	53 (48–57)	0.733
Sex (M:F)	1:28	0:9	> 0.999	1:17	0:9	> 0.999
BMI	23.5 ± 3.8	23.9 ± 4.8	0.839	23.4 ± 3.9	23.9 ± 4.8	0.837
Nodule volume (mL)	35.7 ± 15.4	34.8 ± 11.9	0.872	33.3 ± 9.8	34.8 ± 11.9	0.730
Maximum diameter	5.15 ± 0.81	5.17 ± 0.65	0.948	5.21 ± 0.58	5.17 ± 0.65	0.871
> 1 nodule ablated (%)	10	3	0.705	6 (33)	3 (33)	0.755
Baseline						
TSH	0.82 (0.43–1.06)	0.89 (0.41–1.34)	0.549	0.70 (0.31–1.05)	0.89 (0.41–1.34)	0.341
FT4	18 (15–19)	16 (16–18)	0.328	17 (15–19)	16 (16–18)	0.500
Thyroglobulin	66 (27–274)	172 (118–196)	0.538	103 (24–390)	172 (118–196)	0.679
Anti-TPO positive*	6	4	0.157	5	4	0.386
Anti-Tg positive*	3	1	0.973	2	1	> 0.999
Solid component (%)	100 (90–100)	100 (95–100)	0.655	100 (98–100)	100 (95–100)	0.671
**B**						
3-month VRR (%)	54.8 ± 16.9	59.6 ± 23.8	0.567	55.0 ± 10	59.6 ± 23.8	0.538
6-month VRR (%)	61.4 ± 17.6	71.4 ± 16.7	0.167	57.2 ± 16.1	71.4 ± 16.7	**0.030**
12-month VRR (%)	64.4 ± 20.4	71.2 ± 19.1	0.387	62.3 ± 18.4	71.2 ± 19.1	0.127
Procedural time (s)	1625 (1109–2195)	915 (761–1276)	**0.016**	1791 (1281–2222)	915 (761–1276)	**0.001**
Total energy delivery (J)	70,500 (48,116–100,207)	27,450 (22,815–38,280)	**< 0.001**	62,593 (35,910–92,048)	27,450 (22,815–38,280)	**< 0.001**
Energy per unit volume (J/mL)	2273 (1323–2675)	923 (709–1042)	**< 0.001**	2561 (2153–3102)	923 (709–1042)	**< 0.001**
Ablation time per unit volume (s/mL)	49 (32–70)	31 (23–35)	**0.008**	54 (46–71)	31 (23–35)	**< 0.001**
Compressive symptoms score						
Baseline	40 (30–50)	5 (0–65)	0.265	40 (23–58)	25 (0–51.25)	0.360
12-month	20 (5–30)	0 (0–40)	0.194	20 (0–55)	0 (0–10)	0.243
Cosmetic symptoms score						
Pre-ablation	4 (4–4)	4 (2.3–4)	0.294	4 (4–4)	4 (2.25–4)	0.408
Post-ablation	3 (3–4)	3 (2–3.5)	0.263	3 (3–4)	3 (1–3)	0.211

*VRR* volume reduction rates

Bold = statistically significant (*p* < 0.05)

* 100 U/L

**Fig. 3:**
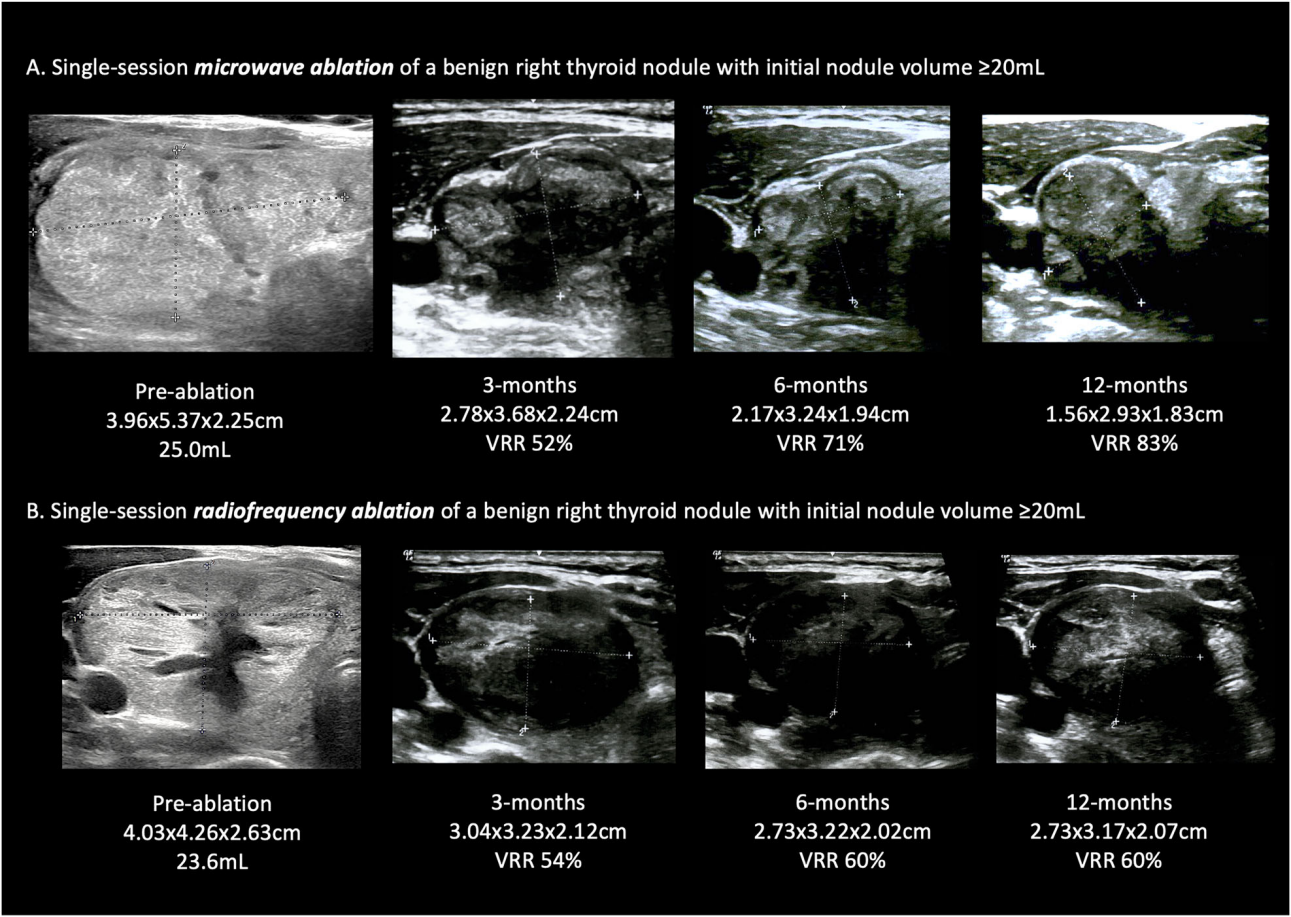
**A** Serial transverse views of a right thyroid nodule of initial volume of 25.0 mL that underwent single-session microwave ablation. The 12-month volume reduction rate was 83%. **B** Serial transverse views of a right thyroid nodule with an initial volume of 23.6 mL that underwent single-session radiofrequency ablation. The 12-month volume reduction rate was 60%

Consistent with the main analysis, both subgroup analyses for large nodules showed MWA required shorter total ablation time, shorter ablation time per unit volume, less total energy delivery, and less energy per unit volume delivery than RFA (*p* < 0.05). Figure 3 shows examples of treatment effect with single-session MWA and RFA to large nodules.

## Discussion

To the best of our knowledge, this is the first study that demonstrates the effect of initial nodule volume on the comparative treatment efficacy of single-session RFA versus single-session MWA for predominantly solid benign thyroid nodules. Maximum nodule diameter and initial nodule volume negatively correlated with the 12-month VRR of single-session RFA, but not the efficacy of single-session MWA. Comparable 12-month VRR was observed between RFA and MWA for smaller nodules (max diameter < 3.5 cm or volume < 20 mL). For larger nodules (max diameter ≥ 3.5 cm or volume ≥ 20 mL), MWA led to greater VRR than RFA.

Previous studies have shown that using RFA to treat large nodules (≥ 20 mL) would require additional treatment sessions to achieve similar VRR as smaller nodules (< 20 mL) [2, 21]. This suggested that initial nodule volume affects the treatment efficacy of a single-session RFA ablation. Despite knowing such, majority of the available literature comparing RFA versus MWA did not match or account for the effect for initial nodule volumes [11–13] (Supplementary Table 6). Cheng et al reported a higher 12-month VRR with RFA (89.6% ± 20% vs 82.5% ± 49.7%, *p* = 0.031) [11]. However, large nodules up to 50–70 mL volume were analyzed together with small nodules. The potential differences in treatment efficacy in large nodules could be diluted by the results from small nodules. Hu et al also reported higher 12-month VRR with RFA (85.4% vs 75.8%, *p* = 0.029) [12], but the unmatched cohort consisted of unequal patient number in both arms, smaller baseline nodule volumes in the RFA group, and different solid component of nodules between RFA and MWA. Cerit et al also reported greater 12-month VRR with RFA, but nodules receiving RFA were much smaller than those receiving MWA (15 mL (2.5–74) vs 40 mL (2–205), *p* < 0.001) [13]. The fundamental differences in baseline characteristics of these unmatched studies did not allow fair comparison between RFA and MWA. Analyzing the outcomes from large nodules together with small nodules would make interpretation and generalizability of results difficult. Therefore, it may be difficult to draw solid conclusions from meta-analyses based on these unmatched studies, without accounting for the differences in baseline characteristics [22, 23].

On the other hand, two retrospective, propensity score-matched studies matched for patient characteristics, nodule size and solid component showed comparable VRR between RFA and MWA, (Yue et al, 83.6% vs 81.6%, *p* = 0.144; Jin et al, 80.1% vs 79.3%, *p* = 0.56) [14, 15], although not all patients reached 12-month follow-up. Chen et al also reported comparable efficacy between RFA and MWA, although baseline nodule volumes were not entirely comparable [24] (Supplementary Table 1). It is important to note that the initial mean/median nodule volume was relatively small for these studies (5.5–10.3 mL), and they suggested that for smaller nodules, RFA and MWA may have comparable efficacy. However, the comparative efficacy of RFA versus MWA in larger nodules remains unknown, and factors that guide the choice of RFA versus MWA remain unclear.

Our results suggested that, while the treatment efficacy of single-session RFA was affected by initial nodule volume and maximum nodule diameter, this effect is less pronounced in single-session MWA. Therefore, larger nodules (predominantly solid) may respond better to single-session MWA than single-session RFA. To allow a fair comparison between RFA and MWA, we performed propensity score matching for baseline patient characteristics and nodule volumes and maximum nodule diameter. Also, the same team of endocrine surgeons, highly experienced with thyroid ablation, performed all procedures with the same technique. We acknowledge that the small sample is a limitation, but our results would prompt future prospective randomized studies to further confirm the relative influence of initial nodule volume on RFA versus MWA efficacy by stratifying comparisons based on such. Also, it would be useful to identify the optimal volume cut-off that led to the greatest difference in treatment efficacy, and hence guide the choice of ablation modality, i.e., MWA over RFA or vice versa. It is worthwhile noting that from our results, both MWA and RFA were safe and allowed ambulatory set-ups.

Several hypotheses may explain how initial nodule volume influences the relative efficacy of RFA versus MWA. First, from our results, MWA achieved a quicker ablation effect than RFA during the procedure (Tables 3–5). For small nodules, the significance of the overall differences in procedural time may be clinically negligible. However, for large nodules, this would lead to significantly longer in procedural times that is clinically significant. Longer procedural times may negatively affect patient’s tolerability of the ablation procedure due to pain and prolonged discomfort, and wearing off of sedation. Also, a long procedural time could be challenging to the operator. These may affect the thoroughness of ablation. Second, a greater volume of overall blood supply or blood flow to larger nodules may affect the efficacy of RFA due to the heat sink effect, whereas this effect would be less pronounced in MWA. Third, large nodules may occupy the whole thyroid lobe, and hence their peripheral zones may be more closely located to the carotid arteries and internal jugular veins, as opposed to smaller nodules, which may be purely intra-thyroidal. Therefore, the ablation effect of RFA on the peripheral zones may again be more affected by the heat sink effect.

There are several limitations of this study. First, the sample size was small, especially for the PSM subgroup comparison in nodules ≥ 20 mL, where microwave ablation (MWA) resulted in significantly greater 6-month VRR, but only numerically greater 12-month VRR (71.2 vs 62.3%, *p* = 0.127). The small sample size of subgroup analyses likely led to insufficient power to detect statistical significance for the 12-month VRR. A similar case was observed in 6-month VRR for nodules with a maximum diameter ≥ 3.5 cm (71.4% vs 62.9%, *p* = 0.083). Nevertheless, subgroup analyses have shown that MWA led to significantly greater 3-month and 12-month VRR for nodules ≥ 3.5 cm, and significantly greater 6-month VRR for nodules ≥ 20 mL, thereby suggesting that MWA may be more efficacious for large nodules. Our exploratory findings from subgroup analyses based on nodule sizes warrant verification with future adequately powered randomized controlled trials. Second, despite greater VRR after MWA for larger nodules, no significant differences were observed in the compressive or cosmetic symptoms between RFA and MWA. It would be valuable to assess how differences in VRR actually correlate with symptoms in future studies. Third, pre-ablation nodule vascularity was not accounted for in the study. Contrast-enhanced ultrasonography (CEUS) was not available in our center. Although color doppler ultrasound (CDUS) may be used to estimate vascularity, solid evidence on its role in the prediction of RFA or MWA outcomes in thyroid ablation was lacking and inconclusive. Majority of studies did not report CDUS vascularity [14–16, 25–28], or formally assess its influence on ablation outcomes [29]. This may be due to the lack of a standardized method to quantify CDUS findings, and its inherent subjective nature and high variability in assessment. Among the limited evidence, the one study that reports CDUS hypervascularity being associated with a 2–3% difference in 12-month VRR after RFA did not adjust its analysis for important, known confounders such as nodule size and solid component [30]. We acknowledge the lack of pre-ablation CEUS or CDUS data on nodule vascularity and hence the assessment of its influence on thyroid ablation outcomes as a limitation of both our study and the literature, and an important area to be addressed in future studies. Fourth, a longer follow-up would be important to compare whether lasting ablation effects, or further VRR could be achieved. Fifth, for large nodules, it is known that a second session of RFA would achieve better and continued volume reduction than a single session [31]. Currently, some centers may recommend more than one session of treatment for large nodules with RFA. The relative comparison of two-session ablation between RFA and MWA remains unknown and would be an important area to be addressed. Finally, the results of the current retrospective study warrant confirmation with prospective, randomized controlled trials.

## Conclusion

Larger nodule diameters and larger initial nodule volumes negatively correlated with the treatment efficacy of single-session RFA for predominantly solid, benign thyroid nodules, but had little influence on the efficacy of single-session MWA. For smaller nodules (maximum diameter < 3.5 cm or volume < 20 mL), single-session RFA and MWA achieved comparable 12-month VRR. For larger nodules (maximum diameter ≥ 3.5 cm or volume ≥ 20 mL), single-session MWA achieved greater volume reduction than RFA. The results of this study prompt future randomized controlled trials to compare RFA versus MWA after stratification by initial nodule volumes or maximum nodule diameters.

## Supplementary information

ELECTRONIC SUPPLEMENTARY MATERIAL

## Abbreviations

Anti-Tg: Anti-thyroglobulin antibodies
Anti-TPO: Anti-thyroid peroxidase antibodies
MWA: Microwave ablation
PSM: Propensity score matching
RFA: Radiofrequency ablation
VRR: Volume reduction rate
