# Supplementary material for: Single-session radiofrequency ablation versus microwave ablation of predominantly solid benign thyroid nodules—a comparison after propensity score matching for initial nodule volumes and diameters
Source: Eur Radiol. 2025 Sep 12;36(3):1783–96. doi: 10.1007/s00330-025-11985-4 (PMC12963161; doi:10.1007/s00330-025-11985-4)
Supplement: Supplementary file 1 — ELECTRONIC SUPPLEMENTARY MATERIAL [file 330_2025_11985_MOESM1_ESM.pdf]

Single-session radiofrequency ablation versus microwave ablation of predominantly solid benign thyroid nodules – a comparison after propensity score matching for initial nodule volumes and diameters

ELECTRONIC SUPPLEMENTARY MATERIAL

Supplementary Table 1. Comparing the number of ablations performed by each of the two surgeons in the tertiary endocrine surgery unit of the cohort (p=0.808)

	RFA (n=142)	MWA (n=66)
Surgeon A	80	36
Surgeon B	62	30

Supplementary Table 2. Intra-Observer Variability of Nodule Volume and Diameter Measurements

Measurement	Intraclass correlation coefficient (95% CI)
Nodule volume	0.999 (0.997–0.999)
Nodule width	0.999 (0.997 - 0.999)
Nodule depth	0.996 (0.993–0.998)
Nodule height	0.999 (0.998–1.000)

Supplementary Table 3a. Comparing volume reduction rates (VRR) for nodule with maximum diameter <3.5cm vs ≥3.5cm; and initial volume <20ml versus ≥20ml in radiofrequency ablation (n=142)

RFA	Maximum nodule diameter <3.5cm (n=72)	Maximum nodule diameter ≥3.5cm (n=70)	P value
3-month VRR (%)	57.8±24.5	56.4±16.2	0.723
6-month VRR (%)	65.8±22.6	64.9±16.1	0.824
12-month VRR (%)	78.2±17.7	68.8±17.3	***0.002***
**RFA**	**Initial volume <20ml (n=113)**	**Initial volume ≥20ml (n=29)**	**P value**
3-month VRR (%)	57.7±21.5	54.8±16.9	0.546
6-month VRR (%)	66.5±19.7	61.4±17.6	0.256
12-month VRR (%)	75.9±16.7	64.4±20.4	***0.002***

Supplementary Table 3b. Comparing volume reduction rates (VRR) for nodule with maximum diameter <3.5cm vs ≥3.5cm; and initial volume <20ml versus ≥20ml in radiofrequency ablation (n=66)

MWA	Maximum nodule diameter <3.5cm (n=48)	Maximum nodule diameter ≥3.5cm (n=18)	P value
3-month VRR (%)	65.0±19.3	60.9±19.8	0.536
6-month VRR (%)	71.4±15.2	70.6±19.0	0.893
12-month VRR (%)	78.0±15.5	77.0±18.2	0.847
**MWA**	**Initial volume <20ml (n=57)**	**Initial volume ≥20ml (n=9)**	**P value**
3-month VRR (%)	59.6±23.8	62.6±19.0	0.734
6-month VRR (%)	70.7±18.2	71.4±16.7	0.923
12-month VRR (%)	78.2±17.1	71.2±19.1	0.259

Supplementary Table 4a. Subgroup analysis of nodules with maximum nodule diameter <3.5cm, comparison of baseline characteristics before and after propensity score matching for age, sex and maximum nodule diameter

	Before propensity score matching			After 1:1 propensity score matching		
	**RFA (n=72)**	**MWA (n=48)**	**P value**	**RFA (n=48)**	**MWA (n=48)**	**P value**
Age	56 (46 – 62)	51 (41 – 58)	0.117	50 (45 – 59)	51 (41 – 58)	0.910
Sex (M:F)	7:65	3:45	0.738	3:45	3:45	>0.999
BMI	23.4±3.3	23.7±5.0	0.775	23.4±3.5	23.7±5.0	0.823
Nodule volume (mL)	5.34±3.21	4.82±3.07	0.375	5.01±3.25	4.82±3.07	0.771
Maximum diameter (cm)	2.52±0.54	2.49±0.63	0.840	2.43±0.58	2.49±0.63	0.615
>1 nodule ablated (%)	49	25	0.082	30	25	0.152
Baseline:<br>TSH<br>FT4<br>Thyroglobulin<br>Anti-TPO positive*<br>Anti-Tg positive^	 0.91 (0.41 –<br>1.77)<br>17 (16 – 19)<br>75 (24 – 270)<br>12<br>14	 1.11 (0.76 –<br>1.44)<br>17 (15 – 18)<br>76 (24 – 192)<br>5<br>5	 0.406<br>0.079<br>0.767<br>0.416<br>0.200	 1.15 (0.55 – 1.89)<br>17 (15 – 18)<br>53 (28 – 256)<br>10<br>8	 1.11 (0.76 –<br>1.44)<br>17 (15 – 18)<br>76 (24 – 192)<br>5<br>5	 0.965<br>0.174<br>0.927<br>0.254<br>0.546
Solid component (%)	100 (100 – 100)	100 (90 – 100)	0.157	100 (100 – 100)	100 (90 – 100)	0.269

Supplementary Table 4b. Subgroup analysis of nodules with maximum nodule diameter <3.5cm, comparison of ablation outcomes before and after propensity score matching for age, sex and maximum nodule diameter

	Before propensity score matching			After 1:1 propensity score matching		
	**RFA (n=72)**	**MWA (n=48)**	**P value**	**RFA (n=48)**	**MWA (n=48)**	**P value**
3-month VRR (%)	57.8±24.5	60.9±19.8	0.555	60.6±22.6	60.9±19.8	0.954
6-month VRR (%)	65.8±22.6	70.6±19.0	0.276	67.6±19.2	70.6±19.0	0.518
12-month VRR (%)	78.2±17.7	77.0±18.2	0.724	77.5±19.7	77.0±18.2	0.907
Procedural time (seconds)	402 (200 – 781)	283 (147 – 372)	***0.004***	398 (199 – 782)	283 (147 – 372)	***0.009***
Total energy delivery (J)	12510 (7092 – 20198)	8475 (4545 – 12698)	***0.001***	12510 (5920 – 19184)	8475 (4545 – 12698)	***0.012***
Energy per unit volume (J/mL)	2586 (2086 – 4216)	1809 (1254 – 2698)	***<0.001***	2672 (2140 – 4408)	1809 (1254 – 2698)	***<0.001***
Ablation time per unit volume (s/mL)	74 (51 – 143)	60 (43 – 88)	***0.036***	76 (52 – 182)	60 (43 – 88)	***0.030***
Compressive symptoms score<br>Baseline<br>12-month	 40 (10 – 55)<br>0 (0 - 15)	 40 (26 – 58)<br>5 (0 – 20)	 0.338<br>0.252	 40 (10 – 51)<br>2.5 (0 – 20)	 40 (26 – 58)<br>5 (0 – 20)	 0.329<br>0.356
Cosmetic symptoms score<br>Pre-ablation<br>Post-ablation	4 (2 – 4)<br>2 (1 – 4)	4 (2.5 – 4)<br>1 (1 – 2)	0.740<br>***0.002***	3.5 (2 – 4)<br>2 (1 – 3.25)	4 (2.5 – 4)<br>1 (1 – 2)	0.550<br>0.013

VRR – volume reduction rates

Eur Radiol (2025) Fung MHM, Luk Y, Lang BHH.

Supplementary Table 5a. Subgroup analysis of nodules with initial volume <20ml, comparison of baseline characteristics before and after propensity score matching

	Before propensity score matching			After 1:1 propensity score matching		
	**RFA (n=113)**	**MWA (n=57)**	**P value**	**RFA (n=57)**	**MWA (n=57)**	**P value**
Age	55 (46 – 63)	51 (41 – 58)	0.086	53 (43 – 60)	51 (41 – 58)	0.357
Sex (M:F)	9:104	5:52	>0.999	6:51	5:52	0.950
BMI	23.1±3.5	23.7±4.7	0.448	23.7±3.7	23.7±4.7	0.951
Nodule volume (mL)	8.6±5.5	6.4±4.8	***0.010***	6.4±4.8	6.4±4.8	0.988
Maximum diameter (cm)	3.05±0.85	2.71±0.78	***0.014***	2.72±0.84	2.71±0.78	0.959
>1 nodule ablated (%)	67	25	0.148	37	25	***0.045***
Baseline:<br>TSH<br>FT4<br>Thyroglobulin<br>Anti-TPO positive*<br>Anti-Tg positive^	0.90 (0.45 –<br>1.53)<br>17 (15 – 18)<br>67 (21 – 201)	1.11 (0.72 –<br>1.48)<br>17 (15 – 18)<br>77 (22 – 228)	0.151<br>0.155<br>0.749	0.88 (0.53 – 1.73)<br>17 (15 – 18)<br>51 (26 – 179)	1.11 (0.72 –<br>1.48)<br>17 (15 – 18)<br>77 (22 – 228)	0.450<br>0.172<br>0.610
Solid component (%)	100 (100 – 100)	100 (90 – 100)	0.775	100 (100 – 100)	100 (90 – 100)	0.395

Supplementary Table 5b. Subgroup analysis of nodules with initial volume <20ml, comparison of ablation outcomes before and after propensity score matching

	Before propensity score matching			After 1:1 propensity score matching		
	**RFA (n=113)**	**MWA (n=57)**	**P value**	**RFA (n=57)**	**MWA (n=57)**	**P value**
3-month VRR (%)	57.7±21.5	62.6±19.0	0.235	59.6±22.4	62.6±19.0	0.530
6-month VRR (%)	66.5±19.7	70.7±18.2	0.220	65.6±21.2	70.7±18.2	0.235
12-month VRR (%)	75.9±16.7	78.3±17.1	0.393	75.8±18.5	78.3±17.1	0.470
Procedural time (seconds)	620 (256 – 979)	328 (169 – 487)	***<0.001***	480 (188 – 833)	328 (169 – 487)	***0.027***
Total energy delivery (J)	18284 (9456 - 35313)	9825 (5070 – 14760)	***<0.001***	12991 (5522 – 25617)	9825 (5070 – 14760)	***0.021***
Energy per unit volume (J/mL)	2585 (2119 – 4053)	1676 (1151 – 2583)	***<0.001***	2558 (1874 – 4295)	1676 (1151 – 2583)	***<0.001***
Ablation time per unit volume (s/mL)	67 (51 – 105)	56 (39 – 85)	***0.028***	70 (49 – 148)	56 (39 – 85)	***0.030***
Compressive symptoms score<br>Baseline<br>12-month	25 (10 – 50)<br>0 (0 - 14)	40 (20 – 50)<br>10 (0 – 17)	0.134<br>0.215	40 (11 – 55)<br>0 (0 – 10)	40 (20 – 50)<br>10 (0 – 17)	0.853<br>0.104
Cosmetic symptoms score<br>Pre-ablation<br>Post-ablation	4 (2 – 4)<br>2 (1 – 3)	4 (3 – 4)<br>1 (1 – 2)	0.874<br>***0.001***	3.5 (2 – 4)<br>2 (1 – 3)	4 (3 – 4)<br>1 (1 – 2)	0.759<br>0.003

VRR – volume reduction rates

Supplementary Table 6. Current evidence comparing single-session radiofrequency ablation (RFA) versus microwave ablation (MWA) for the treatment of benign thyroid nodules

	Type of study	N RFA vs MWA	Baseline nodule volumes RFA vs MWA (mL)	12-month VRR RFA vs MWA (%)	P value	Remarks
**Cheng et al, 2017[11]**	Prospective, unmatched	687 vs 664	7.22 (0.71–52.56) vs 7.72 (0.38–70.16)	RFA > MWA 89.6 ± 20 vs 82.5 ± 49.7	**0.035**	- Unmatched baseline volumes and solid component - large nodules analyzed together with small nodules - Included nodules ≥20% solid
**Hu et al, 2019[12]**	Retrospective, unmatched	72 vs 100	10.7±5.9 vs 13.0±7.9	RFA > MWA 85.4±18.9 vs 75.8±19.4	**0.029**	Unmatched baseline volumes
**Cerit et al., 2023[13]**	Retrospective. unmatchted	37 vs 43	15.6 (2.5-74) vs 40 (2-205)	RFA > MWA 77.9 ± 10.3 vs 65 ± 11.3	**<0.001**	Unmatched baseline volumes and solid component
**Yue et al., 2016[14]**	Retrospective, propensity score matched	102 vs 102	5.7 (3.8–10.3) vs 5.5 (3.5–9.6)	RFA = MWA 83.6 ± 5.2 vs 81.6 ± 8.8	0.144	Not all nodules reached 12-month FU
**Jin et al., 2021[15]**	Retrospective, propensity score matched	289 vs 289	9.2 (5.6 - 18.9) vs 9.2 (5.6- 18.9)	RFA = MWA 80.1 (78.3 - 81.9) vs 79.3 (76.1 - 82.5)	0.56	Results mainly from smaller nodules
**Chen et al., 2024[24]**	RCT	76 vs 76	7.9±7.8 vs 10.3±9.9	RFA = MWA 12-month VRR N/A 24-month VRR 83±24 vs 80±18	0.80	- Unmatched baseline volumes - Results from mainly small nodules - 12-month-VRR not available
